# Influenza exacerbations in an acute and chronic house dust mite ‘asthma’ model

**DOI:** 10.1186/1476-9255-10-S1-P23

**Published:** 2013-08-14

**Authors:** Suzanne M Bal, Lara Ravanetti, Annemiek Dijkhuis, Rene Lutter

**Affiliations:** 1 Department of Experimental Immunology, Academic Medical Centre, University of Amsterdam, Meibergdreef 9, 1105 AZ Amsterdam, The Netherlands; 2 Department of Respiratory Medicine, Academic Medical Centre, University of Amsterdam, Meibergdreef 9, 1105 AZ Amsterdam, The Netherlands; 3 Chiesi Farmaceutici S.p.A., Via Palermo, 26/A - 43100 Parma, Italy

## Rationale

Viral airway infections account for the majority of asthma exacerbations. The underlying mechanisms leading to these viral exacerbations are still not well understood. In both an acute and a chronic ‘asthma’ model using house dust mite (HDM), we investigate the effect of an influenza infection on the inflammatory response.

## Methods

In the acute asthma model, mice were sensitised intranasally with HDM or PBS on three consecutive days and challenged two weeks later. Two days after the challenge the mice were infected intranasally with 10 TCID_50_ influenza (A/PR/8/34) or PBS and sacrificed 4, 8 or 14 days after infection. In the chronic asthma model the mice receive influenza in the 5th week of sensitisation (intranasally, 5 days a week for 5 weeks). In both models we assess(ed) cellular influx by analysing bronchoalveolar lavage fluid (BALF), and cytokines and viral load in lung lysate. PenH is determined as a measure of airway hyperresponsiveness (AHR).

## Results

In the acute asthma model there was a significantly higher influx of eosinophils into the lungs of the HDM-treated mice compared to the HDM/PBS and the PBS/influenza group. Furthermore, in the lungs of HDM-treated mice more IL-5 was produced on day 4 after influenza infection compared to PBS/influenza-infected mice, whereas the IFN-γ production on day 8 was decreased. There was a trend of elevated AHR on day 3 after infection. Currently we implement this exposure in the chronic asthma model to study the effects during a persisting allergic inflammation.

## Conclusion

The acute model shows that in an allergic inflammation a viral infection augments the eosinophillic influx into the lungs and results in a mixed Th1/Th2 response. We are awaiting the data from the chronic model. These models will be used to study mechanisms relevant to exacerbations and corticosteroid insensitivity.

